# Cost-utility and budget impact analysis of laparoscopic bariatric surgery for obesity with Type II Diabetes Mellitus in Thailand

**DOI:** 10.1371/journal.pone.0315336

**Published:** 2024-12-10

**Authors:** Prapaporn Noparatayaporn, Montarat Thavorncharoensap, Usa Chaikledkaew, Panu Looareesuwan, Prapimporn Chattranukulchai Shantavasinkul, Preeda Sumritpradit, Ammarin Thakkinstian

**Affiliations:** 1 Mahidol University Health Technology Assessment (MUHTA) Graduate Program, Bangkok, Thailand; 2 Social and Administrative Pharmacy Division, Department of Pharmacy, Faculty of Pharmacy, Mahidol University, Bangkok, Thailand; 3 Department of Clinical Epidemiology and Biostatistics, Faculty of Medicine Ramathibodi Hospital, Mahidol University, Bangkok, Thailand; 4 Division of Nutrition and Biochemical Medicine, Department of Medicine, Faculty of Medicine Ramathibodi Hospital, Mahidol University, Bangkok, Thailand; 5 Trauma, Acute Care Surgery and Surgical Critical Care Unit, Department of Surgery, Faculty of Medicine Ramathibodi Hospital, Mahidol University, Bangkok, Thailand; Kaohsiung Medical University Chung Ho Memorial Hospital, TAIWAN

## Abstract

Bariatric surgery is another treatment options for patients with obesity, who cannot achieve weight controlled by conservative non-surgical therapy. Although bariatric surgery provides clinical benefits for these patients, it is costly. This study aims to evaluate the cost-effectiveness of bariatric surgery, as compared to nonbariatric surgery, in patients with body mass index (BMI) ≥32.5 kg/m^2^ and type 2 diabetes mellitus (T2DM), and to estimate the budget impact of bariatric surgery in Thailand. Methods: A Markov model was developed to estimate and compare total costs incurred and quality-adjusted life years (QALYs) gained between bariatric surgery and nonbariatric surgery over lifetime horizontal. Analysis was conducted under payer and societal perspectives. Costs and outcomes were discounted at an annual rate of 3%. The outcomes were presented as incremental cost- effectiveness ratio (ICER). Results: Under payer’s perspective, bariatric surgery resulted in higher total lifetime cost (676,658.39 baht vs 574,683.38 baht) and QALYs gained (16.08 QALYs vs 14.78 QALYs), as compared to nonbariatric surgery, resulting in an ICER of 78,643.02 baht/QALY. Similarly, under the societal perspective, bariatric surgery resulted in higher total lifetime cost (1,451,923.83 baht vs 1,407,590.49 baht) and QALYs gained (16.08 QALYs vs 14.78 QALYs), as compared to nonbariatric surgery. Under societal perspective, ICER was estimated at 34,189.82 baht/QALY. A 5-year budget impact analysis indicated that bariatric surgery incurred the total budget of 223,821 million baht. Conclusions: At the cost-effectiveness threshold of 160,000 baht/QALY, bariatric surgery was a cost-effective strategy and should continue to be included in the benefit package for patients with obesity and T2DM.

## Introduction

Bariatric surgery is considered as a treatment option for obese patients, who cannot achieve weight controlled through structured lifestyle changes and pharmacotherapy treatments [1–3]. The clinical effectiveness of bariatric surgery is well established. Previous systematic review studies consistently found that bariatric surgery led to significant weight reduction [4], improvement in comorbidities [5], and diabetes resolution [6], when compared to non-surgical treatment.

Criteria for bariatric surgery varies across regions and ethnical susceptibility to metabolic syndrome. In western countries, bariatric surgery is recommended for patients whose body mass index (BMI) is ≥ 40 kg/m^2^ or ≥ 35 kg/m^2^ with comorbidity [2]. In comparison, the BMI criteria for bariatric surgery is lower for the Asian population [3, 7]. Not only do the criteria for bariatric surgery for Asian and western populations differ [8], but its effect on weight loss and diabetes remission might also vary by ethnicity, where benefits tend to be lesser among the Asian population [9].

Regarding economic evaluations [10], previous systematic review indicated that bariatric surgery was considered as a cost-effective, or even cost-saving, intervention among patients with severe obesity and type 2 diabetes mellitus (T2DM). However, it should be noted that most of the included economic studies were conducted in western high-income countries. The most updated meta-analysis reported that bariatric surgery was also cost-effective for obese patients with T2DM in middle-income countries [11]. However, generalizability of findings may be limited as all four included studies from middle-income countries were conducted under the payer perspective with only one study being conducted in Asia [12]. To date, only four studies on cost-effectiveness of bariatric surgery among obesity with T2DM have been identified in Asia (i.e., China [13, 14], Thailand [12], and Hong Kong [15].

In Thailand, the prevalence of obesity and diabetes mellitus (DM) has increased continuously [16]. Based on 2023 statistics [16–19], the number of patients with BMI ≥32.5 kg/m^2^ and DM aged 18 years and over in Thailand was estimated to be around 457,702. The economic burden of obesity in Thailand was estimated at 12,142 million baht (US$725.3 million), accounting to 0.13% of Thailand’s Gross Domestic Product [20]. According to the Thai Society for Metabolic and Bariatric Surgery’s guideline on bariatric surgery, it is recommended for patients whose BMI ≥ 37.5 kg/m^2^, or ≥ 32.5 kg/m^2^ with comorbidity [1].

An economic evaluation study in Thailand revealed that bariatric surgery was cost-effective in obesity with T2DM, as compared to usual care [12]. The study focused on the effectiveness of bariatric surgery through five health states in the Markov model (i.e., DM remission, improved DM, persistent DM, uncontrolled DM, and dead). The study obtained key short-term outcome of bariatric surgery (i.e., 1-year DM remission) from retrospective analysis of a tertiary hospital in Thailand. In comparison, such outcome in the usual care group was obtained from the literature review from western country. Additionally, utility values were derived from western countries. Furthermore, the previous Thai study considered payer perspective rather than the societal perspective, which is recommended by Thailand’s health technology assessment guideline. Finally, the budget impact analysis (BIA) of implementing bariatric surgery has not been conducted.

This study, therefore, aims to assess the cost-utility and BIA of bariatric surgery for Thai obesity with T2DM. Real-world data of obesity with T2DM was used to estimate the effect of bariatric surgery on diabetes remission and relapse including the risks of developing complications. Input parameters including cost, utility, and transitional probabilities of developing DM complications among obese patients with T2DM were obtained from primary data collection in Thailand. Both payer and societal perspectives were examined. Although bariatric surgery has been included under the Thai universal coverage scheme since January 2021, the study finding will provide real-world evidence to confirm the surgery’s value for money.

## Materials and methods

Cost-utility analysis was conducted using the Markov model to estimate and compare total costs incurred and quality-adjusted life years (QALYs) gained between bariatric surgery and nonbariatric surgery. Two types of bariatric surgery (i.e., laparoscopic Roux-en-Y gastric bypass (LRYGB) and laparoscopic sleeve gastrectomy (LSG)) were considered as they are the most commonly performed methods in Thailand [1]. The nonbariatric surgery group was defined as usual therapeutic treatments for obesity and T2DM. The analysis was performed using a lifetime horizon with a one-year cycle length based on payer and societal perspective. A discounting rate of 3% per year was applied to both costs and outcomes, as recommended by Thailand’s health technology assessment guideline [21]. To determine whether bariatric surgery was cost-effective, an incremental cost-effectiveness ratio (ICER) was compared to the willingness-to-pay threshold of 160,000 baht per QALY [22].

### Target population

The target population were Thai patients with BMI ≥32.5 kg/m^2^ and T2DM, aged 40 years or older, who had not yet developed any of the following complications: myocardial infarction (MI), stroke, congestive heart failure (CHF), and chronic kidney disease (CKD). The BMI criteria of ≥32.5 kg/m^2^ was used as it was the recommended criteria for bariatric surgery by the Thai Society for Metabolic and Bariatric Surgery Consensus Guideline [1]. The target population age was based on the average age of patients who had undergone bariatric surgery at Ramathibodi hospital, a tertiary teaching hospital in Bangkok.

### Model structure and assumption

**Fig 1:**
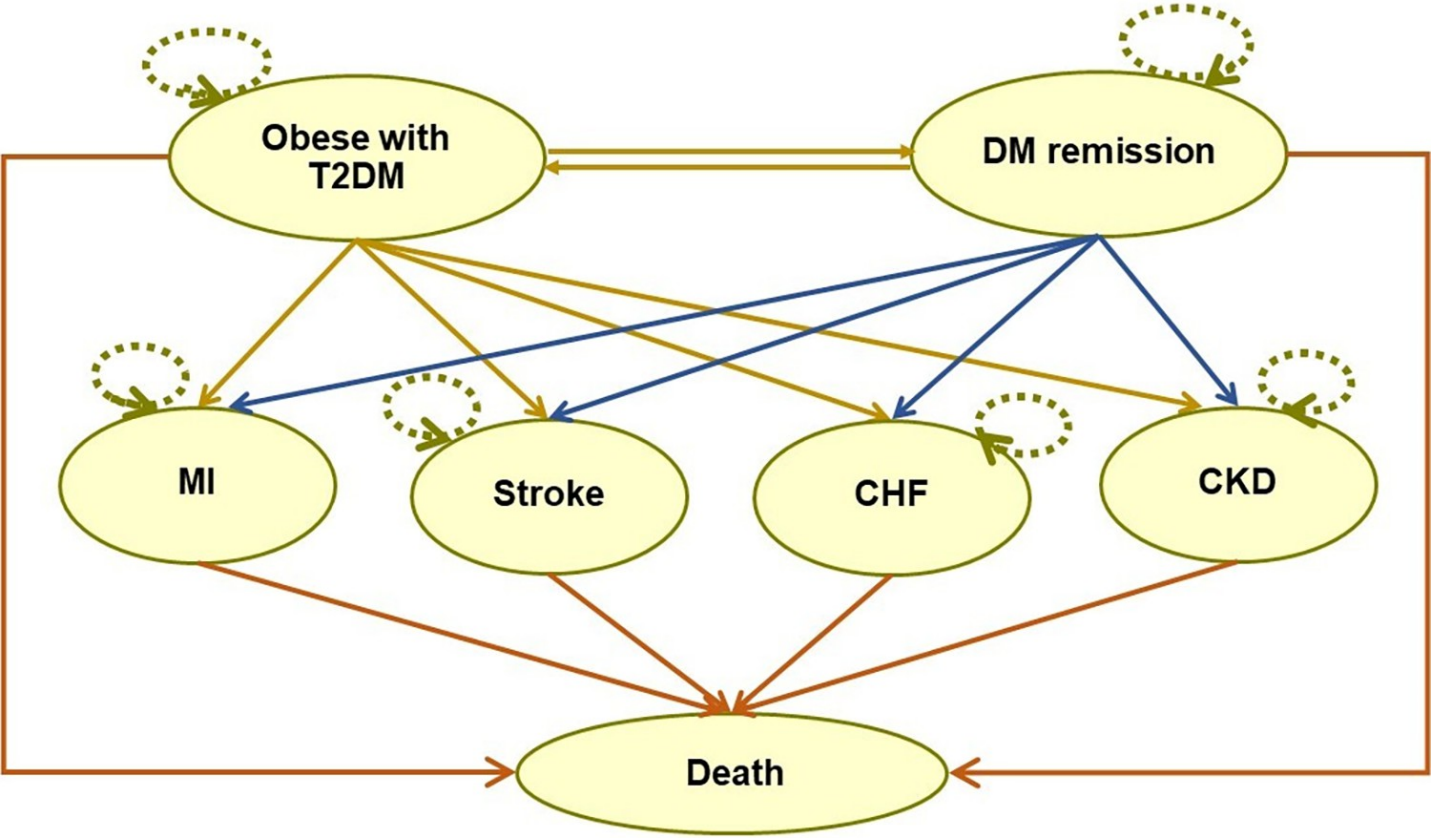
Markov model.

Our Markov model (Fig 1) consisted of seven health states: obese with T2DM, DM remission, MI, stroke, CHF, CKD, and death. The model was adapted from an Australian study [23] and the IMS CORE Diabetes Model [24].

The model shows that obesity with T2DM patients at the initial state were treated either with bariatric surgery or nonbariatric surgery. After treatment, only some patients could achieve DM remission. However, DM relapse could occur among patients with initial DM remission. As the disease progressed, each patient type (i.e., with DM remission or non-remission) could develop obesity/T2DM-related complications including macrovascular complications (i.e., MI, CHF, and stroke) and microvascular complication (i.e., CKD).

As for the model assumption, it was assumed that: 1) patients who were treated with bariatric surgery would undergo surgery only one time, and 2). whenever patients developed complications (i.e., MI, stroke, CHF, CKD), both groups of patients (i.e., bariatric surgery and nonbariatric surgery group) had the same costs of treatment and utility score.

### Input parameters

#### Transitional probabilities

The transitional probabilities were estimated from a real-world cohort of T2DM; the Department of Clinical Epidemiology and Biostatistics, Faculty of Medicine Ramathibodi [25, 26]. Patients were classified as having T2DM if they met any of the following criteria: 1) diagnosed with ICD-10 code E11 (non-insulin-dependent diabetes mellitus) + HbA1c ≥ 6.5% (48 mmol/mol) or FBG ≥ 126 mg/dL on two separates test; 2) HbA1c ≥ 6.5% (48 mmol/mol) or FBG ≥ 126 mg/dL on two separates test + received antidiabetic drug; or 3) diagnosed with ICD-10 code E11 + received antidiabetic drug. These T2DM patients were eligible if they met the following criteria: 1) aged ≥ 18 years old: 2) BMI ≥ 32.5 kg/m^2^: and 3) received treatment at Ramathibodi hospital during 2010–2019. Patients with existing complications (i.e., MI, stroke, CHF, CKD) at their entry of the T2DM cohort were excluded. Eligible patients were then classified into bariatric surgery group if they received bariatric surgery (ICD-9: 4382, 4431, 4438) while the others were classified into the usual care group. Note that patients in the bariatric group were excluded if they had a revisional procedure.

Finally, a total of 2,139 patients met the eligibility criteria. Of these, 30 patients received bariatric surgery. The mean age at diagnosis of T2DM of the patients in the bariatric surgery group was 39.9 years old while the mean age at the surgery was 40.5 years old. In contrast, the mean age at diagnosis of the nonbariatric surgery group was 52.2 years old. The mean (SD) BMI of the nonbariatric and bariatric groups were 36.69 (4.37) kg/m^2^ and 43.31 (6.94) kg/m^2^, respectively. Transitional probabilities of DM remission and relapse by treatment groups were then estimated based on this real-world cohort. Multivariate analyses were employed to adjust for possible confounders. Patients in the cohort were classified as having DM remission if: their HbA1c < 6.5% (<48 mmol/mol) or fasting plasma glucose (FPG) < 126 mg/dL (<7.0 mmol/L) or the estimated HbA1c calculated from continuous glucose monitoring values <6.5% without any diabetic medications for at least three months [27]. Furthermore, transitional probabilities of DM-related complications among those without DM remission regardless of treatment group was also obtained. Due to the limited number of patients in the DM remission group, transitional probabilities of DM-related complications among this group were calculated from the probabilities in the non-remission group and relative risks (RRs) from previous meta-analyses [28, 29]. Note that 19 (63.3%) and 402 (19.1%) individuals in the bariatric surgery and nonbariatric surgery groups achieved DM remission, respectively. In the non-remission group and relapse group (n = 2,076), 351 (16.91%) individuals reported having the complications after entering the cohort.

To determine age-specific mortality rate among obesity with T2DM patients, the RR of mortality associated with obesity with T2DM [30] was applied to the age-specific mortality probability of Thailand’s population [31]. As previous meta-analysis revealed that improvement of glycemic control was not associated with all-cause mortality [29], probability of death from all-cause mortality among DM remission was assumed to be similar to those without remission. Annual probability of death of patients with T2DM [30, 31], stroke [32], CHF [33], MI [34], and CKD [35] were estimated from literature reviews. Annual transitional probabilities were summarized in Table 1 and S1 Table.

**Table 1:** Input parameters.

Variable	Distribution	Mean	SE	Source
**Transitional probabilities**				
Obese with T2DM to DM remission in non-BS group	Beta	0.0156	0.0047	Ramathibodi T2D cohort
Obese with T2DM to DM remission in BS group (the 1^st^ year)	Beta	0.4333	0.13	Ramathibodi T2D cohort
Obese with T2DM to DM remission in BS group (the 2^nd^ year)	Beta	0.2941	0.0882	Ramathibodi T2D cohort
Obese with T2DM to DM remission in BS group (the 3^rd^ year)	Beta	0.0833	0.025	Ramathibodi T2D cohort
Obese with T2DM to DM remission in BS group (the 4^th^ year and onward)	Beta	0.0156	0.0047	Ramathibodi T2D cohort
DM remission relapse to DM in non-BS group	Beta	0.0708	0.0212	Ramathibodi T2D cohort
DM remission relapse to DM in BS group	Beta	0.0107	0.0032	Ramathibodi T2D cohort
Obese with T2DM to MI	Beta	0.0008	0.0002	Ramathibodi T2D cohort
Obese with T2DM to stroke	Beta	0.0031	0.0009	Ramathibodi T2D cohort
Obese with T2DM to CHF	Beta	0.002	0.0006	Ramathibodi T2D cohort
Obese with T2DM to CKD	Beta	0.0066	0.002	Ramathibodi T2D cohort
DM remission to MI	Beta	0.0007	0.0002	Ramathibodi T2D cohort, [29]
DM remission to stroke	Beta	0.0027	0.0008	Ramathibodi T2D cohort, [29]
DM remission to CHF	Beta	0.002	0.0006	Ramathibodi T2D cohort, [28]
DM remission to CKD	Beta	0.0049	0.0015	Ramathibodi T2D cohort, [29]
Death from MI	Beta	0.032	0.0096	[34]
Death from stroke	Beta	0.0494	0.0148	[32]
Death from CHF	Beta	0.325	0.0975	[33]
Death from CKD	Beta	0.1098	0.0329	[35]
**Direct medical cost**				
***Cost of obesity treatment in Non-BS group***				
Cost of treatment in Obese with T2DM group (baht/year)	Gamma	30,504.77	1,066.81	Hospital database
Cost of treatment in DM remission group (baht/year)	Gamma	15,437.65	5,893.15	Hospital database
***Cost of obesity treatment in BS group***				
Cost of pre-operative management and surgery (baht/operation)	Gamma	279,899.62	6,865.92	Hospital database
Cost of treatment among obese with T2DM group in the 1^st^ year (baht/year)	Gamma	36,536.72	7,987.91	Hospital database
Cost of treatment among obese with T2DM group in the 2^nd^ year (baht/year)	Gamma	30,114.01	8,517.57	Hospital database
Cost of treatment among obese with T2DM group in the 3^rd^ year and onward (baht/year)	Gamma	30,589.87	8,298.79	Hospital database
Cost of treatment in DM remission group in the 1^st^ year (baht/year)	Gamma	16,518.51	2,483.20	Hospital database
Cost of treatment in DM remission group in the 2^nd^ year (baht/year)	Gamma	16,941.94	1,299.82	Hospital database
Cost of treatment in DM remission group in the 3^rd^ year and onward (baht/year)	Gamma	9,085.91	1,200.82	Hospital database
***Cost of treatment for co-morbidity***				
Cost of MI treatment in the 1^st^ year (baht/year)	Gamma	119,212.12	47,059.54	Hospital database
Cost of MI treatment in other years (baht/year)	Gamma	28,612.87	41,451.42	[36]
Cost of stroke treatment (baht/year)	Gamma	46,501.62	12,464.44	Hospital database
Cost of CHF treatment (baht/year)	Gamma	59,300.76	10,902.97	Hospital database
Cost of CKD treatment (baht/year)	Gamma	53,085.23	18,647.41	Hospital database
**Direct non-medical cost**				
***Non-BS & BS group***				
Cost of accommodation in the 1^st^ year (baht)	Gamma	5,598.86	1,571.74	Interview
Cost of home renovation in the 1^st^ year (baht)	Gamma	1,159.09	734.09	Interview
Traveling cost of OP visit (baht/visit)	Gamma	766.36	88.54	Interview
Food cost of OP visit (baht/visit)	Gamma	275.45	57.23	Interview
Hotel cost of OP visit (baht/visit)	Gamma	73.86	32.63	Interview
Cost of informal care at OP visit (baht/visit)	Gamma	81.85	25.23	Interview
Traveling cost of IP visit (baht/admission)	Gamma	1,062.50	849.36	Interview
Food cost of IP visit (baht/admission)	Gamma	425	265.75	Interview
Cost of informal care at IP visit (baht/admission)	Gamma	4,242.86	2,034.44	Interview
Cost of informal care in obese with T2DM group (baht/year)	Gamma	12,396.90	8,649.82	Interview
Cost of hiring assistance among obese with T2DM (baht/year)	Gamma	22,307.69	9,962.56	Interview
Cost of informal care among DM remission group (baht/year)	Gamma	4,933.46	4,933.46	Interview
Cost of hiring assistance among DM remission group (baht/year)	Gamma	14,448.98	5,813.21	Interview
Treatment cost outside hospital (other hospital, drug, supplementary food) among obese with DM remission (baht/year)	Gamma	2,348.98	422.75	Interview
***Non-BS group***				
Treatment cost outside hospital (other hospital, drug, supplementary food) among obese with T2DM in non-BS group (baht/year)	Gamma	1,396.77	547.32	Interview
***BS group***				
Treatment cost outside hospital (other hospital, drug, supplementary food) among obese with T2DM in BS group (baht/year)	Gamma	10,645.88	1,781.32	Interview
**Direct non-medical cost of comorbidity**				
MI (baht/year)	Gamma	51,785.00	1,822.00	[37]
Stroke (baht/year)	Gamma	63,159.45	18,947.84	[38]
CHF (baht/year)	Gamma	33,942.00	6,912.00	[39]
CKD (baht/year)	Gamma	168,863.00	50,658.90	[40]
**Utility**				
Obese with T2DM without complication	Beta	0.83	0.02	Interview
DM remission without complication	Beta	0.97	0.01	Interview
MI in obese with T2DM/DM remission	Beta	0.79	0.01	Interview, [41, 42]
Stroke in obese with T2DM/DM remission	Beta	0.6	0.02	Interview, [41, 43]
CHF in obese with T2DM/DM remission	Beta	0.75	0.03	Interview, [41, 44]
CKD in obese with T2DM/DM remission	Beta	0.7	0.03	Interview, [41, 45]

BS: bariatric surgery; CHF: congestive heart failure; CKD: chronic kidney disease; DM: diabetes mellitus; IP: in-patient; MI: myocardial infarction; OP: out-patient**;** T2DM: type 2 diabetes mellitus

### Cost

Direct medical costs, which included cost of diagnosis, treatment, and follow-up for obesity, T2DM, and obesity/DM-related complications as well as cost of pre- and peri-operative for bariatric surgery were derived from a real-world cohort of T2DM, as previous mentioned.

Direct non-medical costs of patients with obesity and T2DM either with or without DM remission were collected from face-to-face interviews. This sample size was calculated using the following formula [46], where, Z_1-α/2_ = 1.96, α = 0.05, σ represents standard deviation, and ε represents margin of error.


$$n=\frac{{Z^{2}}_{1-\alpha /2}\times \sigma^{2}}{\mu^{2}\varepsilon^{2}}$$

According to the review, the mean of direct non-medical costs of DM remission and non-remission from the previous studies were AUS$ 411 [23] and US$75.53 [14], respectively. When assuming that the SD was approximately 50% of the mean, and the margin of error was set at 0.15, the sample size required for each group (i.e., DM remission group, and non-DM remission group) was 43.

To collect data for direct non-medical costs, Thai patients with BMI ≥32.5 kg/m^2^ and T2DM were invited to interview if they: 1) received treatment at a nutrition clinic for ≥ 3 months or received bariatric surgery ≥ 3 month; 2) were aged 18 years or above; and 3) were willing to participate in the interview. The interviews were performed by trained nurse and nutritionist between April 2021 to February 2022. Due to COVID-19, data were collected from 39 patients without DM remission and 49 patients with DM remission, respectively. To calculate costs of informal care, the number of hours providing informal care was multiplied by cost per hour, estimated from Gross National Income [47, 48]. Direct non-medical costs of each complication were obtained from the literature reviews [37–40].

All costs were calculated and adjusted to the currency year 2023, using consumer price index [49]. Moreover, a ratio of cost-to-charge (1.63) was used for adjusting charge from hospital database to cost [50]. Information on the costs used is summarized in Table 1.

### Utility

Utility of patients with obesity and T2DM, with and without DM remission were collected from face-to-face interviews using a Thai version of the European Quality of life (EuroQol) 5-Dimensions 5-Levels (EQ-5D-5L) [51]. Permission to use the questionnaire was obtained from the EuroQol Research Foundation. Utilities for each complication were obtained from literature reviews [42–45]. To estimate the utility of patients with obesity and T2DM with complication, a disutility method was adopted, using the population norm for Thailand [41]. Note that the patients who were interviewed for utility were the same as those interviewed for direct non-medical costs.

### Sensitivity analysis

The impacts of variables on uncertainty of cost-utility analysis were examined using one-way sensitivity analysis and probabilistic sensitivity analysis (PSA). For PSA, Monte Carlo simulation was generated to randomly select a value of each variable for 1,000 times. A gamma distribution was assigned for cost parameters while a beta distribution was assigned for the probability and utility parameters. The PSA results were presented by a cost-effectiveness plane and acceptability curves.

### BIA

BIA was performed to estimate the financial consequence of bariatric surgery in Thailand across a time horizon of 5 years (2023–2027) from the health care system perspective. According to Metabolic and Bariatric Surgery Consensus Guideline, the patients undergoing bariatric surgery should be between 18 and 65 years [1]. Therefore, the target population of BIA were patients with BMI ≥ 32.5 kg/m^2^ and T2DM aged 18–65 years.

According to the Department of Provincial Administration, the total https://dopa.go.th/main/web_indexpopulation aged between 18 and 65 years in Thailand in April 2023 was 45,094,408 [18]. Prevalence of BMI ≥ 30 kg/m^2^ in each gender and age (18–65 years) groups derived from the National Health Examination Survey (2019–2020) ranged across 5.5% - 19.6% [16]. Then, the population number with BMI ≥ 30 kg/m^2^ aged 18–65 years were estimated to be 6,359,953 (see S2 Table). A previous Thai study [19] found that 4.0%, 0.8%, and 0.1% of the Thai population met the criteria for class I (BMI = 30.0–34.9 kg/m^2^), class II (BMI = 35.0–39.9 kg/m^2^), and class III obesity (BMI ≥ 40.0 kg/m^2^), respectively. Therefore, a population with BMI ≥ 32.5 kg/m^2^ was accounted for 59.2% of the population with BMI ≥ 30 kg/m^2^. Thus, the number of population with BMI ≥ 32.5 kg/m^2^ was estimated to be 3,764,102. As 11.1% of patients with obesity have T2DM [17], the number of population with BMI ≥32.5 kg/m^2^ with T2DM aged 18–65 years was estimated to be 417,865.

To estimate budget impact, 417,865 was used to represent the number of target population in 2023. The number of target population in 2024–2027 was estimated based on the incidence of T2DM among patients with obesity [52]. With the eight-year incidence of T2DM among men and women with BMI ≥32 kg/m^2^ of 22.52% and 18.03%, respectively [52], the annual incidence of T2DM were 3.1% in men and 2.5% in women. Given that the number of population aged 18–65 years with BMI ≥ 32.5 kg/m^2^ (no T2DM) was 3,346,237, the new cases of T2DM among the population were estimated to be 90,744. Note that the new cases of obese patients with T2DM were assumed to remain constant during 2024–2027.

### Ethical considerations

This study was approved by the Human Research Ethics Committee, Faculty of Medicine Ramathibodi Hospital, Mahidol University (COA. MURA2021/140). Written informed consent was obtained from all patients before the interview. The database was accessed after the study was approved by the Human Research Ethics Committee.

## Results

### Base case analysis

Under payer perspective, bariatric surgery resulted in higher total lifetime cost and QALYs gained as compared to nonbariatric surgery, i.e., 676,658.39 baht vs 574,683.38 baht and 16.08 QALYs vs 14.78 QALYs, respectively, which resulted in an ICER of 78,643.02 baht/QALY (see Table 2).

**Table 2:** Cost-effectiveness results.

Comparator strategies	Lifetime cost (Baht)*	Life years (LY)	QALYs	ICER (Baht/LY)	ICER (Baht/QALY)
Payer perspective					
Bariatric surgery	676,658.39	17.92	16.08	838,836.38	78,643.02
Non-surgery	574,683.38	17.8	14.78	-	-
Societal perspective					
Bariatric surgery	1,451,923.83	17.92	16.08	364,681.67	34,189.82
Non-surgery	1,407,590.49	17.8	14.78	-	-

ICER: incremental cost effectiveness ratio; LY: Life year, QALY: Quality-adjusted life year

*The exchange rate of 35.06 baht equal 1 US$

Similarly, the social perspective result was consistent with payer perspective in that bariatric surgery resulted in higher total lifetime costs (1,451,923.83 baht vs 1,407,590.49 baht) and QALYs gained (16.08 QALYs vs 14.78 QALYs). An ICER from societal perspective was estimated at 34,189.82 baht/QALY gained (see Table 2). Under the cost-effectiveness threshold of 160,000 baht/QALY gained, bariatric surgery was considered cost-effective under both perspectives.

### Sensitivity analysis

**Fig 2:**
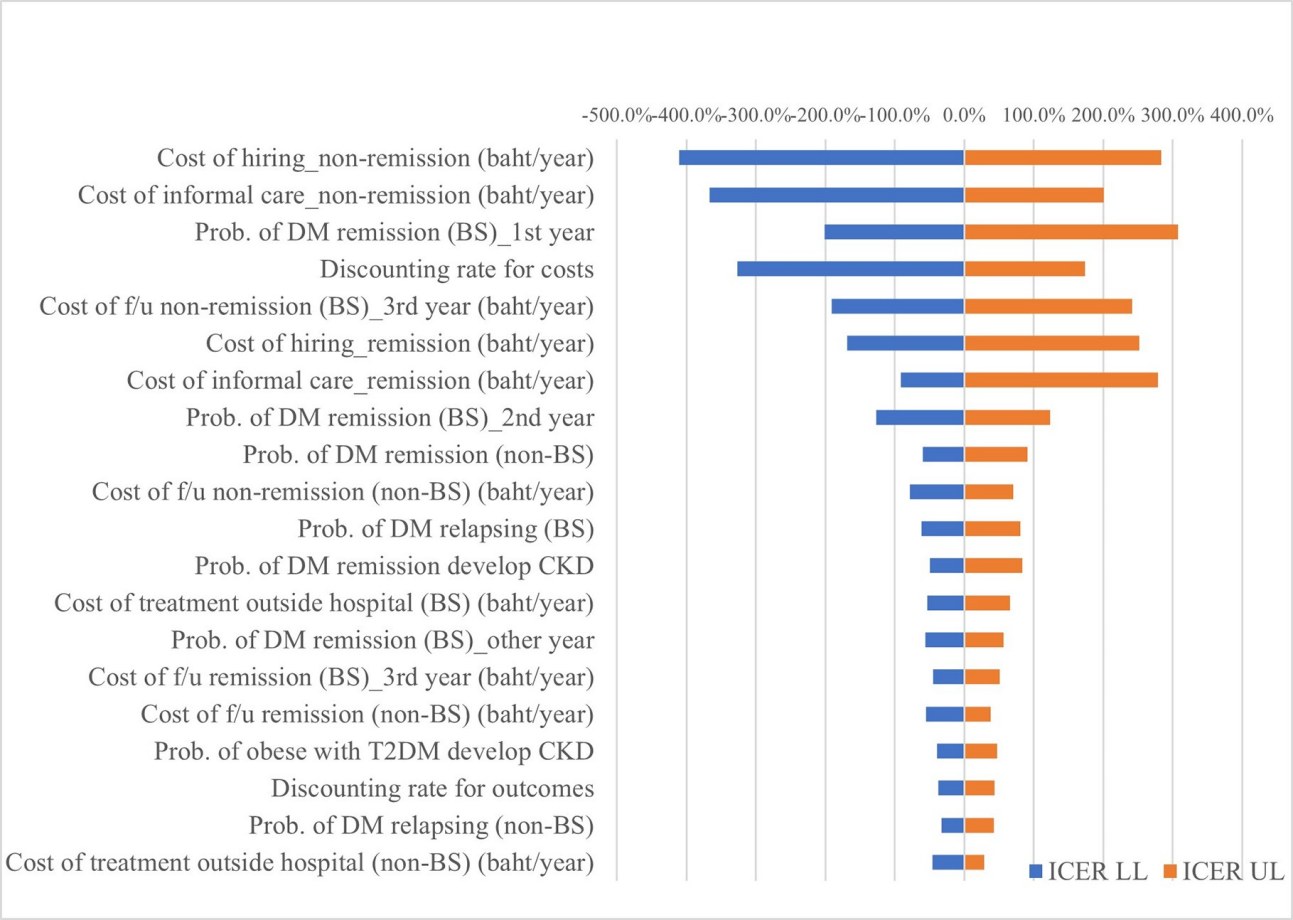
One-way sensitivity analysis results under societal perspective.

One-way sensitivity analysis was performed and presented in a Tornado diagram (Fig 2). From a societal perspective, the three most influential parameters that affected the ICER were cost of hiring assistance among those without DM remission, cost of informal daily care among those without DM remission, and probability of DM remission during the first year among the bariatric group.

**Fig 3:**
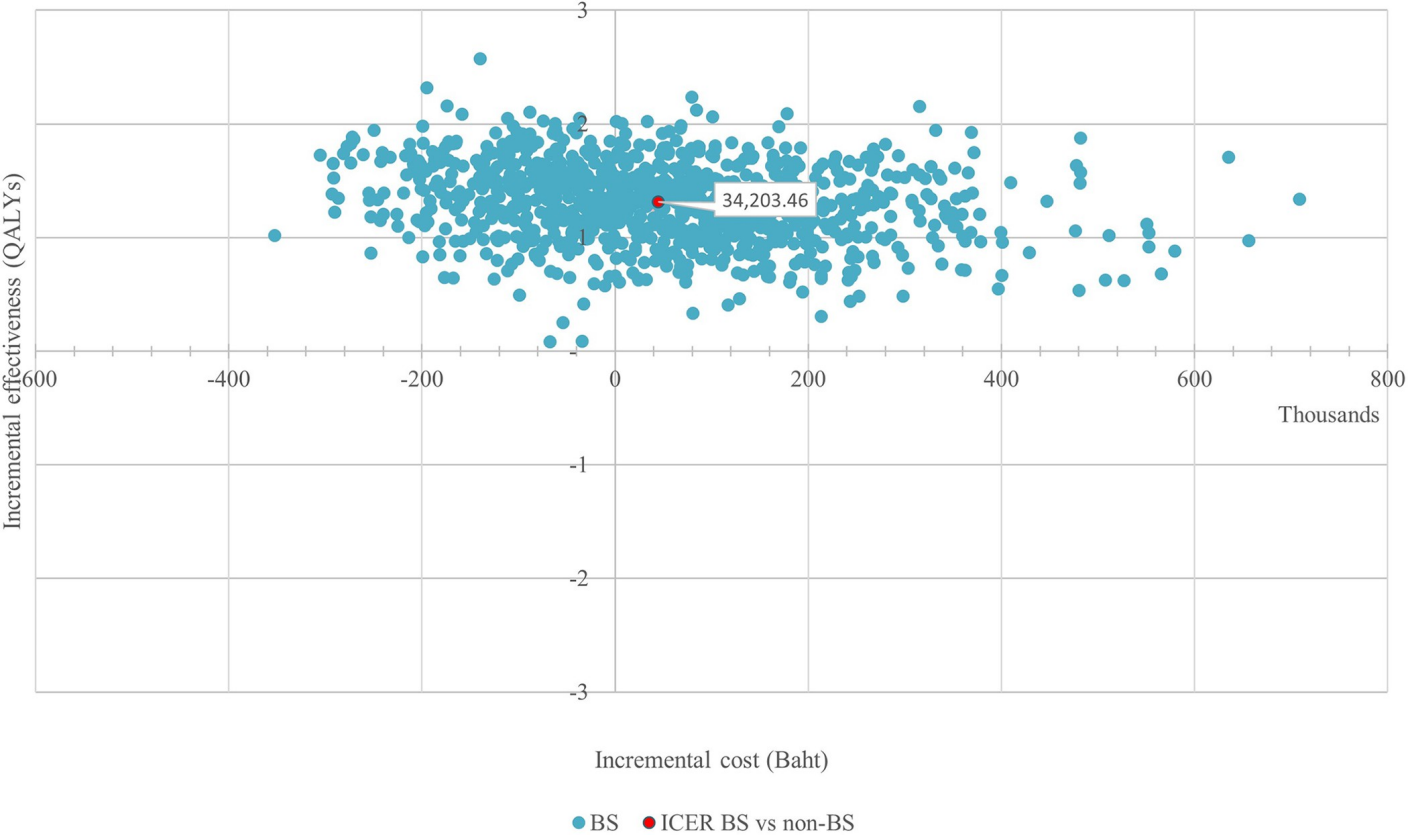
Cost-effectiveness plane under societal perspective.

**Fig 4:**
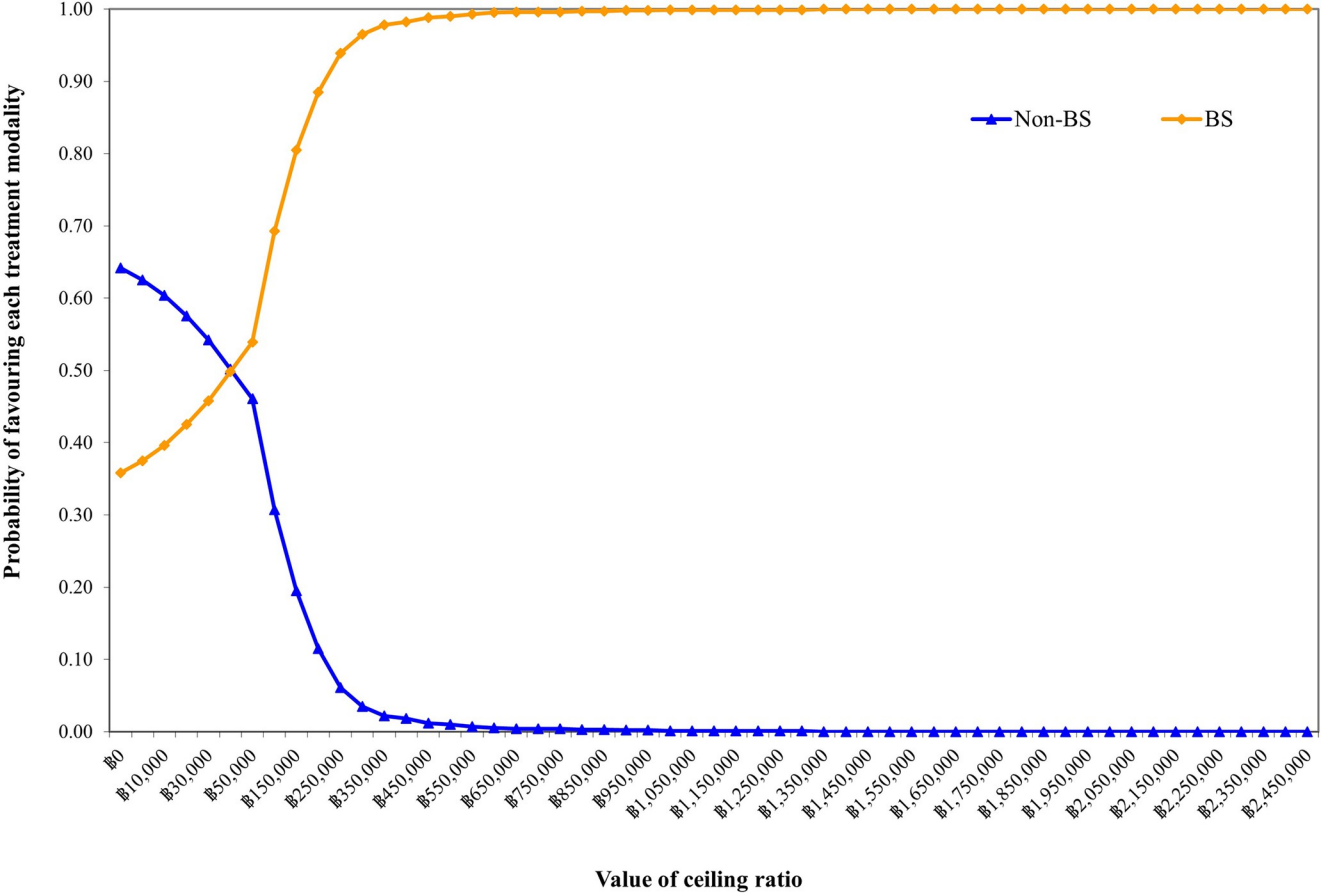
Cost-effectiveness acceptability curve under societal perspective.

For PSA, approximately 42% of the simulations under societal pespective revealed that bariatric surgery resulted in less total lifetime costs but higher QALYs. As shown in Fig 3, the ICER from the simulation was estimated at 34,203.46 baht/QALY (incremental cost = 44,891.77 baht, incremental QALY = 1.31). In Fig 4, bariatric surgery had about 83% chance to be a cost-effective option under a societal perspective at a current threshold of 160,000 baht/QALY. Results of uncertainty analysis under payer perspective were further provided in S1–S3 Figs.

#### BIA

For the bariatric surgery group, preoperative and operative costs accounted for the largest proportion (about 88%) of the total costs in the first year, whereas cost of treatment accounted for a small proportion. Compared to nonbariatric surgery, bariatric surgery incurred an incremental budget of 223,821 million baht during the 5 years. Details on 5-year total budget and their components were further provided in S3 Table.

## Discussion

We conducted a cost-utility analysis of bariatric surgery based on the key information obtained from real-world cohort of Thai patients with obesity and T2DM. The results revealed that bariatric surgery was a cost-effective intervention for Thai patients with BMI ≥ 32.5 kg/m^2^ and T2DM, who have not yet developed complications under both payer and societal perspectives with the ICER of 78,643.02 and 34,189.82 baht/QALY, respectively.

Our result was similar to that of previous meta-analysis [11], which found that bariatric surgery was considered a cost-effective intervention among patients with obesity and T2DM under a payer perspective. This could be due to the benefits of bariatric surgery in DM remission, which resulted in the reduction of incidences of obesity-related complications leading to a decrease in medical utilization (i.e. reduction in the number of medication, out-patient visits, and hospitalizations) and improvement in utility [53]. For the societal perspective, bariatric surgery was considered cost-effective with the lower ICER, when compared to the payer perspective. This is likely because increasing the probability of DM remission, bariatric surgery also led to higher reduction in direct non-medical costs.

While almost all of the previous economic evaluation studies were conducted on patients with obesity and T2DM, whose BMI ≥ 35 kg/m^2^ [11], the BMI criteria in our study was relatively lower (BMI ≥ 32.5 kg/m^2^). The findings from our study also indicated that bariatric surgery was still cost-effective. This finding complements those conducted among patients with T2DM, whose BMI < 35 kg/m^2^ [12–14, 54].

Our findings were also consistent with the previous cost-effectiveness study conducted in Thailand (ICER = 26,908 baht/QALY; 2017 value) [12]. However, our study’s model and sources of input parameters differed [12]. While most of the input parameters in the previous studies were derived from western countries, most of the parameters in our study were from Thailand. Regarding costs, the cost of bariatric surgery in our study was higher than that the previous study (280,000 bath in 2023 vs 150,000 baht in 2017). Note that cost of bariatric surgery in our study also included preoperative management. Estimated transition probability from obesity with T2DM to DM remission in our study was lower than that of previous study. In the previous study [12], 1-year probability of DM remission after surgery was 84% whereas our study was 43% in the first year, 29% in the second year, 8% in the third year, and 2% in the fourth year onward. Nevertheless, it was found that the remission rate at 1-year in the previous study [12] also seemed to be higher than that of other studies. According to the recent systematic review, remission rate at the first year was 47% and 57% for patients undergoing RYGB and SG, respectively [55]. In the studies that were conducted in Southeast Asia [56, 57], the remission rates in Thailand and Malaysia were 67% and 54.2%-59%, respectively. Nevertheless, several factors including age of onset and duration of T2DM alongside BMI were associated with remission rate [58]. Additionally, the remission rate could also vary depending on the definition of remission, which slightly differed across studies [56, 57, 59]. Therefore, direct comparison of remission rates across studies should be made with caution.

Since January 2021, bariatric surgery has been included in the health benefit package for Thailand’s universal coverage scheme. This study provides important evidence to support the current policy decision regarding bariatric surgery coverage. Nevertheless, our findings indicate that bariatric surgery incurs substantial 5- year budget impact for the government. Under the current limited capacity and resources to perform bariatric surgery, efforts should be made to improve the accessibility to such service and to prioritize groups of patients, who will benefit the most from the surgery. Further economic evaluation studies of bariatric surgery conducted in patients with different DM duration and different types of comorbidity are warranted to assist prioritization processes.

Our study has several strengths. While DM remission from bariatric surgery may depend on ethnicity [9], our study adopted real-world cohort data in the country to estimate the impact of bariatric surgery on DM remission and relapse. Additionally, most input parameters including cost, utility, and transitional probabilities among obesity with T2DM and DM complications were derived from primary data collection in the Thai context. While most past studies were conducted under payer perspective, with several not adopting a lifetime perspective [10, 11], this study considered both perspectives over lifetime horizon. As the cost of bariatric surgery was quite high in the early years [1] while its benefits could be life-long, analysis under lifetime horizon will be more appropriate than short-term time horizon.

Nevertheless, our study has limitations. Firstly, our analyses might not capture the wider benefits of bariatric surgery such as increased attachment to labor market. Additionally, only four obesity-related complications were considered. If other complications such as angina pectoris, transient ischemic attack, and peripheral arterial diseases were included, bariatric surgery would have been even more cost-effective [60]. Furthermore, due to the limited data on LSG, cost-effectiveness by each type of bariatric surgery (i.e., LSG, LRYGB) could not be performed. However, previous meta-analyses found that there was no significant difference in clinical outcomes such as DM [5, 55] and hypertension remission rates [5] between RYGB and SG. Regarding utility, the utility scores of those receiving RYGB and SG were similar [61]. Therefore, both types of bariatric surgery should have similar cost-effectiveness profiles, as noted in a previous study [14]. It should also be noted that our probabilities of developing complication were estimated from the cohort of newly diagnosed T2DM with obesity, therefore, could be lower than those of other studies [25]. Furthermore, our direct non-medical costs were based on the information collected from the sample of adults (age ≥18 years old), who were receiving care at one tertiary hospital in Bangkok. Although the mean (SD) age of our sample was 44.83 (11.57) years old, the generalizability of this information to adults age ≥40 years old across country should be made with caution. In addition, utility data were derived from patients receiving treatment at one tertiary hospital in Bangkok, which may not be representative of patients nationwide. Cost-utility analysis employed data from multi-centers are warranted. Finally, similar to the limitation mentioned in most previous studies that information on long-term effect of bariatric was scarce [10, 11], effect of bariatric surgery in our study was only based on the analysis of one hospital’s 10-year database. Future research which incorporates long-term effectiveness and safety data of bariatric surgery from real-world evidences are needed.

## Conclusions

Based on the real-world cohort data on the impact of bariatric surgery on DM remission and relapse, bariatric surgery was a cost-effective intervention for patients with obesity and T2DM in the Thai context under payer and societal perspectives. Our findings provide crucial evidences to support the current coverage decision of bariatric surgery in the country. Based on the cost-effectiveness evidence, bariatric surgery should continue to be a treatment for patients with a BMI of 32.5 kg/m^2^ and T2DM, especially among those who have not yet developed comorbidity. Given the substantial budget impact and the limited health care resources and capacity to provide bariatric surgery, efforts should be made to improve accessibility to such service and to prioritize the group of patients who will benefit the most from bariatric surgery.

## Supporting information

S1 Fig Tornado diagram from payer perspective. (PDF)

S2 Fig Cost-effectiveness plane from payer perspective. (PDF)

S3 Fig Cost-effectiveness acceptability curve from payer perspective. (PDF)

S1 Table Input parameters. (PDF)

S2 Table Prevalence of Thai population with BMI ≥ 30 kg/m^2^. (PDF)

S3 Table Budget impact analysis for bariatric surgery over 5-year period. (PDF)
