# Supplementary material for: Cost-utility and budget impact analysis of laparoscopic bariatric surgery for obesity with Type II Diabetes Mellitus in Thailand
Source: PLoS One. 2024 Dec 10;19(12):e0315336. doi: 10.1371/journal.pone.0315336 (PMC11630598; doi:10.1371/journal.pone.0315336)
Supplement: S2 Fig — (PDF) [file pone.0315336.s002.pdf]

Supporting information

Cost-Utility and Budget Impact Analysis of Laparoscopic Bariatric Surgery for Obesity with Type II Diabetes Mellitus in Thailand

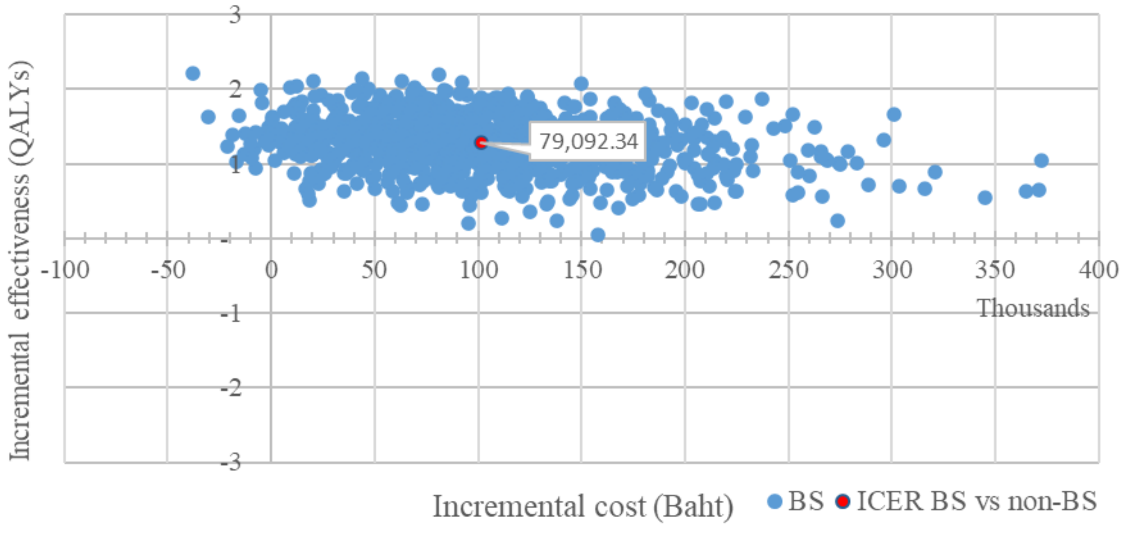

S2 Fig Cost-effectiveness plane from payer perspective
