# Supplementary material for: Cost-utility and budget impact analysis of laparoscopic bariatric surgery for obesity with Type II Diabetes Mellitus in Thailand
Source: PLoS One. 2024 Dec 10;19(12):e0315336. doi: 10.1371/journal.pone.0315336 (PMC11630598; doi:10.1371/journal.pone.0315336)
Supplement: S1 Table — (PDF) [file pone.0315336.s004.pdf]

Supporting information

Cost-Utility and Budget Impact Analysis of Laparoscopic Bariatric Surgery for Obesity with Type II Diabetes Mellitus in Thailand

S1 Table Input parameters

Variable	Distribution	Mean	SE	Source
**Number of hospital visits**				
Non-BS group				
Number of OP visit in obese with T2DM group (visit/year)	Gamma	2.94	0.04	Hospital database
Number of IP visit in obese with T2DM group (admission/year)	Gamma	0.01	0.004	Hospital database
Number of OP visit in DM remission group (visit/year)	Gamma	2.52	0.23	Hospital database
Number of IP visit in DM remission group (admission/year)	Gamma	0.05	0.05	Hospital database
BS group				
Number of OP visit pre-BS surgery (visit/operation)	Gamma	4.77	0.324	Hospital database
Number of OP visit in obese with T2DM group in the 1st year (visit/year)	Gamma	4.13	0.401	Hospital database

Number of OP visit in obese with T2DM group in the 2nd year (visit/year)	Gamma	3.5	0.535	Hospital database
Number of OP visit in obese with T2DM group in the 3rd year and onward (visit/year)	Gamma	2.86	0.404	Hospital database
Number of IP visit in obese with T2DM group in the 1st year (admission/year)	Gamma	1	0	Hospital database
Number of IP visit in obese with T2DM in group in the 2nd year and onwards (admission/year)	Gamma	0	0	Hospital database
Number of OP visit in DM remission group in the 1st year (visit/year)	Gamma	3.29	0.474	Hospital database
Number of OP visit in DM remission group in the 2nd year (visit/year)	Gamma	2.43	0.228	Hospital database
Number of OP visit in DM remission group in the 3rd year and onward (visit/year)	Gamma	2	0.302	Hospital database
Number of IP visit in DM remission group (admission/year)	Gamma	0	0	Hospital database

BS: bariatric surgery; non-BS: non-bariatric surgery; T2DM: type 2 diabetes mellitus; OP: out-patient**;** IP**:** in-patient
