# Supplementary material for: Cost-utility and budget impact analysis of laparoscopic bariatric surgery for obesity with Type II Diabetes Mellitus in Thailand
Source: PLoS One. 2024 Dec 10;19(12):e0315336. doi: 10.1371/journal.pone.0315336 (PMC11630598; doi:10.1371/journal.pone.0315336)
Supplement: S2 Table — (PDF) [file pone.0315336.s005.pdf]

Supporting information

Cost-Utility and Budget Impact Analysis of Laparoscopic Bariatric Surgery for Obesity with Type II Diabetes Mellitus in Thailand

S2 Table prevalence of Thai population with BMI ≥ 30 kg/m^2

Age (years)	Male [1]	Female [1]	Prevalence of male with BMI ≥ 30 kg/m^2 [2]	Prevalence of female with BMI ≥ 30 kg/m^2 [2]	Male with BMI ≥ 30 kg/m^2	Female with BMI ≥ 30 kg/m^2
18-29	5,431,881	5,212,879	13.9%	14.6%	755,036	761,088
30-44	7,241,066	7,231,675	12.2%	19.6%	883,419	1,417,414
45-59	7,234,096	7,921,534	8.5%	18.4%	614,907	1,457,570
60-65	2,222,028	2,599,249	5.5%	13.4%	122,216	348,303
Total	22,129,071	22,965,337			2,375,578	3,984,375

References

1. Official statistics registration systems. Population and housing statistical data divided by age. The Bureau of Registration Administration. [Available from: https://stat.bora.dopa.go.th/stat/statnew/statMenu/newStat/home.php [Accessed May 5, 2023].
2. Ekaphalakorn V. Report of Thai public health survey by physical examination No. 6, Nonthaburi: Health System Research Institute; 2021.
