# Supplementary material for: Cost-utility and budget impact analysis of laparoscopic bariatric surgery for obesity with Type II Diabetes Mellitus in Thailand
Source: PLoS One. 2024 Dec 10;19(12):e0315336. doi: 10.1371/journal.pone.0315336 (PMC11630598; doi:10.1371/journal.pone.0315336)
Supplement: S3 Table — (PDF) [file pone.0315336.s006.pdf]

Supporting information

Cost-Utility and Budget Impact Analysis of Laparoscopic Bariatric Surgery for Obesity with Type II Diabetes Mellitus in Thailand

S3 Table Budget impact analysis for bariatric surgery over 5-year period

Year	Non-bariatric surgery group (Million Baht)	Bariatric surgery group (Million Baht)		Incremental budget (Million Baht)
		Cost of surgery	Cost of treatment	
1	15,515	142,359	18,583	145,427
2	18,240	25,399	15,716	22,874
3	20,892	25,399	15,379	19,886
4	23,501	25,399	16,418	18,316
5	26,070	25,399	17,988	17,317
Total	104,218	243,956	84,083	223,821
